# Pyroptosis: mechanism and therapeutic strategies with intervertebral disc degeneration

**DOI:** 10.1038/s12276-025-01630-x

**Published:** 2026-01-22

**Authors:** Wang Wu, Zhangrong Cheng, Xianglong Chen, Pengzhi Shi, Anran Zhang, Haiyang Gao, Wenbo Wu, Yukun Zhang

**Affiliations:** Department of Orthopedics, Union Hospital, Tongji Medical College, Huazhong University of Science and Technology, Wuhan, China

**Keywords:** Biomaterials, Biomaterials - cells

## Abstract

Intervertebral disc degeneration (IDD) is a major cause of low back pain, characterized by a complex interplay of inflammation, extracellular matrix degradation and various modes of cell death. Among these, pyroptosis, a highly inflammatory form of programmed cell death, has recently emerged as a critical pathogenetic mechanism. This review systematically elaborates on the role of pyroptosis in IDD, detailing its activation via the canonical and noncanonical inflammasome pathways in response to oxidative stress, mechanical load and metabolic disturbances. Here we highlight how pyroptosis synergizes with other pathological processes, creating a vicious cycle that accelerates disc degeneration. Furthermore, we critically evaluate promising therapeutic strategies that target pyroptosis, including small molecule inhibitors, biological agents, stem cell-derived extracellular vesicles and innovative biomaterial-based delivery systems designed to overcome the challenges of the avascular disc microenvironment. Finally, we discuss the translational potential and future directions of antipyroptosis therapies, proposing an integrated approach for managing IDD.

## Inflammation and cell death fuel intervertebral disc decline

Intervertebral disc degeneration (IDD) is a condition that affects the spine, causing pain and reduced quality of life. The study explores how a process called pyroptosis contributes to IDD. Pyroptosis involves the release of inflammatory substances that damage cells and tissues. Researchers reviewed existing studies to understand this process better. The study highlights that pyroptosis is triggered by stress factors like oxidative stress and mechanical stress on the spine. These stresses activate a protein complex called the NLRP3 inflammasome, leading to cell death and inflammation. The researchers used various methods, including reviewing past research and analyzing biological pathways, to understand how pyroptosis affects IDD. The findings suggest that targeting pyroptosis could be a potential treatment strategy for IDD. By inhibiting the NLRP3 inflammasome, it may be possible to reduce inflammation and slow down disc degeneration.

This summary was initially drafted using artificial intelligence, then revised and fact-checked by the author.

## Introduction

IDD is a complex degenerative disease of the musculoskeletal system, seriously threatening human health and quality of life^1^. As the primary pathological basis of LBP, the pathogenesis of IDD is highly complex, involving multiple biological levels of interaction^2^. At the biomechanical level, the long-term uneven mechanical stress on the intervertebral disc (IVD) leads to the gradual degeneration of extracellular matrix (ECM) structure and function^3^. Metabolic abnormalities, such as insulin resistance, oxidative stress and chronic inflammation, further accelerate the degeneration process of IVD tissue^4^.

Pyroptosis, as a new form of PCD, plays a crucial role in the pathogenesis of IDD. Unlike apoptosis, pyroptosis is a unique form of PCD with a more complex molecular mechanism. In the pathological progression of IDD, pyroptosis mainly exerts its effect through activating the NLRP3 inflammasome. When IVD cells are exposed to oxidative stress, mitochondrial dysfunction or DNA damage, it triggers the assembly and activation of the NLRP3 inflammasome. After activation, the NLRP3 inflammasome recruits caspase-1, which cleaves inflammatory factors such as IL-1β and IL-18. These factors directly damage IVD cells and amplify the inflammatory cascade reaction through positive feedback^5^. Pyroptosis leads to cell membrane rupture and releases a large amount of inflammatory factors and intracellular contents, further exacerbating local inflammation and tissue damage^6^. Studies have found that pyroptosis is closely related to endoplasmic reticulum stress, abnormal autophagy and oxidative stress. Mitochondrial dysfunction, calcium ion homeostasis imbalance, and excessive production of reactive oxygen species (ROS) caused by oxidative stress are all key factors triggering pyroptosis^7^. While the involvement of pyroptosis in IDD pathogenesis is increasingly recognized, several critical questions remain contentious. First, the hierarchical importance of pyroptosis relative to other well-established pathways such as apoptosis and dysfunctional autophagy is unclear; are they parallel events, or does one initiate a cascade leading to others? Second, the trigger-specificity of pyroptosis activation—whether it is primarily driven by mechanical stress, metabolic derangements or infection in sterile environments warrants further elucidation. Furthermore, translating these mechanistic insights into therapies faces the formidable challenge of the avascular, harsh disc microenvironment, which impedes drug delivery and efficacy. This Review summarizes the current knowledge on pyroptosis in IDD and critically examine these unresolved controversies and assess the translational viability of targeting this pathway.

## Histological and pathological mechanisms of IVD and IDD

### Structure and function of IVD

The IVD is located between adjacent vertebrae and connects them into a continuous semi-rigid column. The IVD plays multiple vital roles in the spine. The IVD comprises the annulus fibrosus (AF), the nucleus pulposus (NP) and the cartilage endplates (CEP). The AF is a tough, ring-shaped structure surrounding the IVD, composed of 15–25 collagen fiber layers crisscrossing at a 30–60° angle. Under external pressure, the AF deforms in response to NP displacement, thereby absorbing and distributing spinal loads. Within physiological limits, this mechanism dissipates forces exerted on the NP, vertebrae and spinal structure. The NP, enclosed by the AF, is centrally located within the IVD. It comprises a gelatinous matrix rich in NP cells (NPCs), proteoglycans, type II collagen fibers and high water content. This unique composition provides essential compressive resistance and hydraulic cushioning, critical for spinal mobility and mechanical load distribution. The CEP is a thin hyaline-like cartilage layer covering the superior and inferior aspects of the NP. Since blood vessels are confined to the outer AF, the CEP serves as the primary pathway for nutrient and oxygen permeation to the inner disc cells^8^.

### Pathologic mechanisms of IDD

**Fig. 1:**
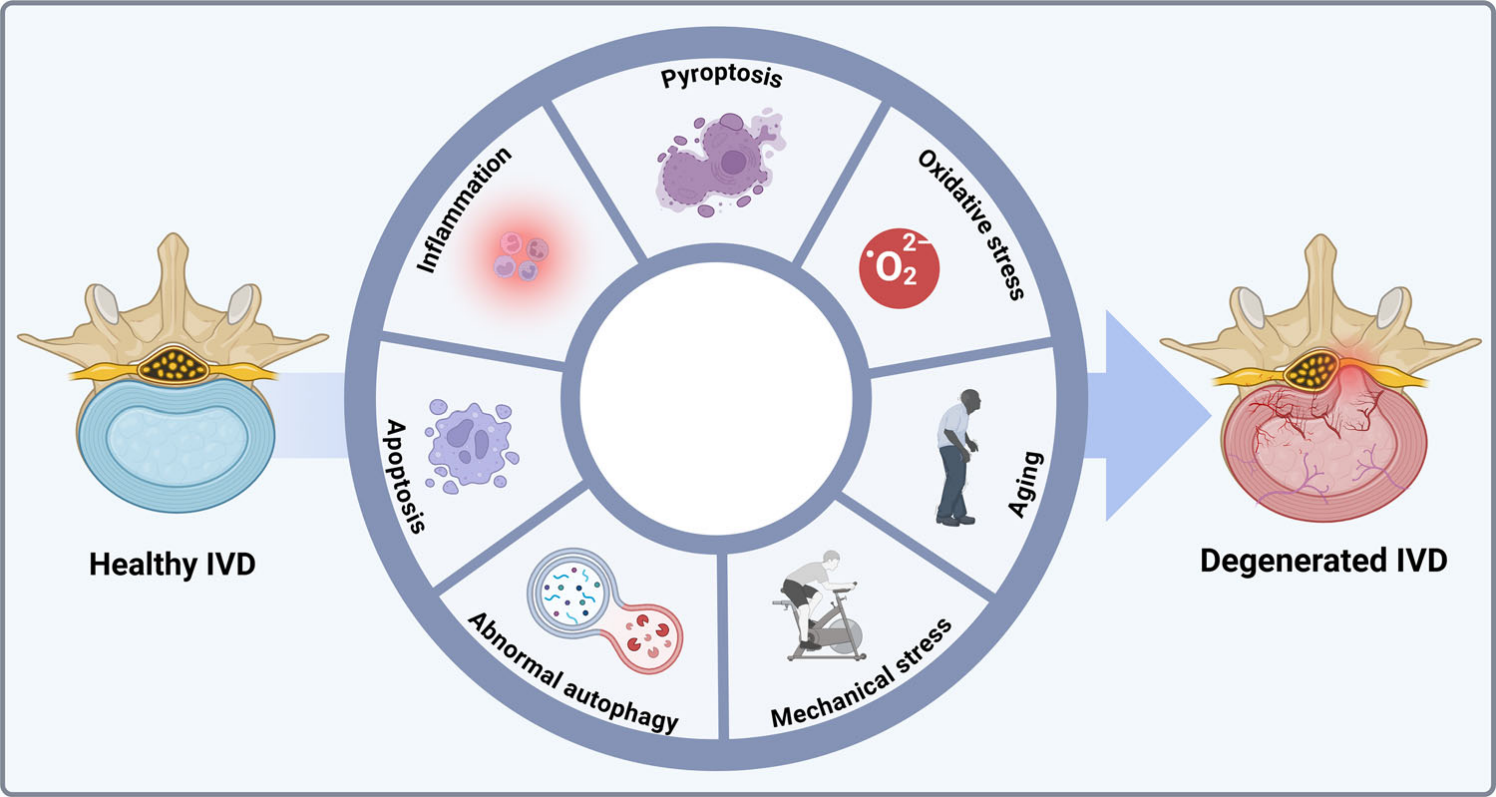
Multiple pathologic mechanisms of IDD. (Created by Biorender).

A healthy IVD maintains cellular and ECM homeostasis, allowing it to withstand normal movement and load. However, ECM degradation accelerates once inflammatory pathways are activated, and pro‑inflammatory cytokines flood the disc space. Excessive apoptosis and pyroptosis diminish the viable cell population, while abnormal autophagy prevents the effective removal of damaged organelles and proteins. Concurrent oxidative stress, driven by ROS, further injures disc cells and age-related cell function declines, making the tissue unable to repair itself. Repeated mechanical stress compounds these effects, generating microdamage that the disc can no longer heal. These intertwined processes break down disc structure and function, ultimately resulting in IDD (Fig. 1).

#### Molecular and cellular mechanisms of IDD

##### Inflammation

Inflammation is important in IDD. Pro-inflammatory cytokines, including TNF, IL-1β, IL-17 and IL-8 accumulate markedly in degenerated discs, forming a self-amplifying cascade that sustains inflammatory activation. These cytokines critically disrupt ECM homeostasis by upregulating matrix metalloproteinases (MMPs) and a disintegrin and metalloproteinase with thrombospondin motifs (ADAMTS), the key catabolic enzymes, which dominate ECM degradation, accelerate structural disintegration and promote IDD progression^9^.

##### Oxidative stress

As the largest avascular structure in the human body, IVD is characterized by hypoxia, acidity, hyperosmolarity and nutrient deficiency in its microenvironment. Oxidative stress plays a crucial role in this environment, stemming from the excessive production of ROS caused by mitochondrial dysfunction, and the antioxidant defense mechanism is insufficient to address this imbalance^10^. The excessive accumulation of ROS is closely related to IDD. Research has shown that IDD is closely related to oxidative stress and affects the aging and degeneration of NPCs through the ROS–Hippo–p53 pathway^11^.

##### Autophagy

Autophagy is an intrinsic regulatory cellular process that prevents the accumulation of harmful molecules and protects cells by clearing damaged proteins and dysfunctional organelles^12^. However, autophagy dysfunction is closely related to IDD. The impaired autophagy flow in NPC caused by lysosomal dysfunction promotes the development of IDD. Restoring lysosomal function in NPCs alleviates damage and prevents mechanical overload-induced IDD, indicating the potential therapeutic value of targeting autophagy in IDD treatment^13^. In IVD, adenosine 5‘-monophosphate-activated protein kinase (AMPK) and mammalian target of rapamycin (mTOR) are the main autophagy regulatory factors. Research has shown that BMSC Exos promotes autophagy and reduces apoptosis by inhibiting the Akt–mTOR pathway^14^. On the contrary, miR-654-5p targets the ATG7 gene, activates the PI3K–AKT–mTOR signaling pathway to inhibit autophagy, promotes ECM degradation and exacerbates IDD^15^. In summary, mTOR activation as an autophagy inhibitor is considered a potential strategy to hinder and delay the progression of IDD.

##### Cell senescence

Cell senescence is a state of permanent cell cycle arrest, resulting in decreased proliferation and differentiation abilities, accompanied by physiological function decline, leading to ECM degradation and associated with chronic diseases^16^. The accumulation of senescent cells is considered the key factors in age-related diseases. In IDD, oxidative stress, DNA damage and mitochondrial damage are the main pro-aging factors^17^. The aging process of NPC affects IDD through inflammatory responses and oxidative stress^18^. Research has shown that circKIF18A reduces oxidative stress-induced senescence of NPCs by inhibiting MCM7 degradation^19^. In summary, oxidative stress and inflammatory factors accelerate senescence through multiple pathways, reduce active cells in IVDs and promote the progression of IDD.

##### Apoptosis

Apoptosis is an important pathological process in IDD, involving three main mechanisms: exogenous death receptor pathway, endogenous mitochondrial pathway and endoplasmic reticulum stress pathway^20^. Researchers found that Fas and Fas ligand levels were significantly elevated in IVD tissues. Moreover, death receptor 4 (DR4) was strongly expressed in degenerated herniated discs, and both DR4 and DR5 were associated with apoptosis of IVD cells and IDD. These findings suggest that death receptor-mediated apoptosis contributes to IDD^21^.

##### Pyroptosis

Pyroptosis contributes to IDD by activating inflammasomes and releasing inflammatory mediators such as IL-1β and IL-18^5^. In IDD, factors such as ECM degradation products and oxidative stress act as damage-associated molecular patterns (DAMPs), triggering NLRP3 inflammasome activation^22^. This leads to gasdermin D (GSDMD) cleavage, pore formation in the cell membrane and the release of pro-inflammatory cytokines, which exacerbate local inflammation, promote ECM degradation and drive further pyroptosis in a destructive cycle^23^. Oxidative, mechanical and metabolic stresses have been shown to induce NLRP3-mediated pyroptosis in NPCs and AF cells, highlighting the pathway as a promising therapeutic target for mitigating IDD progression^24,25^.

##### The interaction between pyroptosis and other mechanisms in IDD

IDD is unlikely to be attributable to a single form of cell death. Rather, a complex interplay between pyroptosis, apoptosis, and autophagy dysfunction forms a self-amplifying degenerative network. For instance, while pyroptosis-derived cytokines indirectly trigger apoptosis in bystander cells via death receptor signaling, the key interaction is the caspase-3-mediated cleavage of gasdermin E (GSDME), which acts as a molecular switch that converts an apoptotic signal into inflammatory pyroptotic cell lysis^26^. Conversely, impaired autophagy fails to clear damaged organelles such as mitochondria, leading to ROS accumulation and NLRP3 activation, thereby potentiating pyroptosis^27^. This creates a vicious cycle: pyroptosis-derived inflammation can further suppress autophagic flux and promote apoptosis^28^. A self-reinforcing cycle links senescence and pyroptosis: pyroptosis induces senescence via persistent IL-1β exposure and NF-κB activation, while senescent cells reciprocally amplify pyroptosis through the persistent inflammatory signals of the SASP^29^. With inflammation, pyroptosis is both a major consequence and a primary driver; the process is initiated by inflammatory stimuli and, upon execution, releases a massive wave of additional cytokines, thereby perpetuating a chronic inflammatory state that fuels all other degenerative pathways in a vicious cycle^30^.

The relative dominance of each pathway may depend on the disease stage and the nature of the insult. Apoptosis might dominate in early, low-grade degeneration under sustained mechanical stress, while a more intense inflammatory or oxidative insult could push the balance toward pro-inflammatory pyroptosis.

The interaction between pyroptosis and other PCDs, such as necroptosis and ferroptosis, remains unclear in IDD. Pyroptosis and necroptosis are both pro-inflammatory, but their relationship is unclear. They may be activated in parallel by common upstream signals^31^. Ferroptosis, driven by iron-dependent lipid peroxidation, could theoretically generate oxidative DAMPs such as oxidized lipids that activate the NLRP3 inflammasome, thereby initiating pyroptosis. Conversely, pyroptosis-induced inflammation might alter iron metabolism or antioxidant defenses, sensitizing cells to ferroptosis^32^.

#### Mechanical changes in IDD

During the IDD process, substantial changes occur in the mechanical structure of IVD, affecting its biomechanical properties and functions^3^. The dehydration and degeneration of the NP reduce its elasticity and buffering capacity, impairing its ability to disperse external forces and increasing the fragility of the disc^33^. During degeneration, the collagen fibers of the AF break and become disordered, reducing strength and stability, increasing the risk of tearing and leading to NP protrusion or displacement^34^. The calcification and hardening of the endplate cartilage hinder the transport of nutrients and accelerate the degeneration of the NP and AF^35^. Disc height loss results from NP dehydration and AF degeneration, causing abnormal vertebral contact and wear that further exacerbates degeneration^36^. In addition, the IVD may undergo morphological changes such as bulging, protrusion or herniation, which can compress surrounding nerve structures and cause pain and functional impairment^37^.

The IDD involves various biological processes, with pyroptosis emerging as a key focus. Pyroptosis contributes to IDD under inflammatory, oxidative and mechanical stress, accelerating degeneration and potentially exacerbating the condition by promoting angiogenesis and neuro-ingrowth^38^. Therefore, a deeper understanding of pyroptosis in IDD helps reveal its pathology and identify potential therapeutic targets. The following section will explore its molecular mechanisms and specific roles.

## Pyroptosis and IDD

### Overview of pyroptosis

**Fig. 2:**
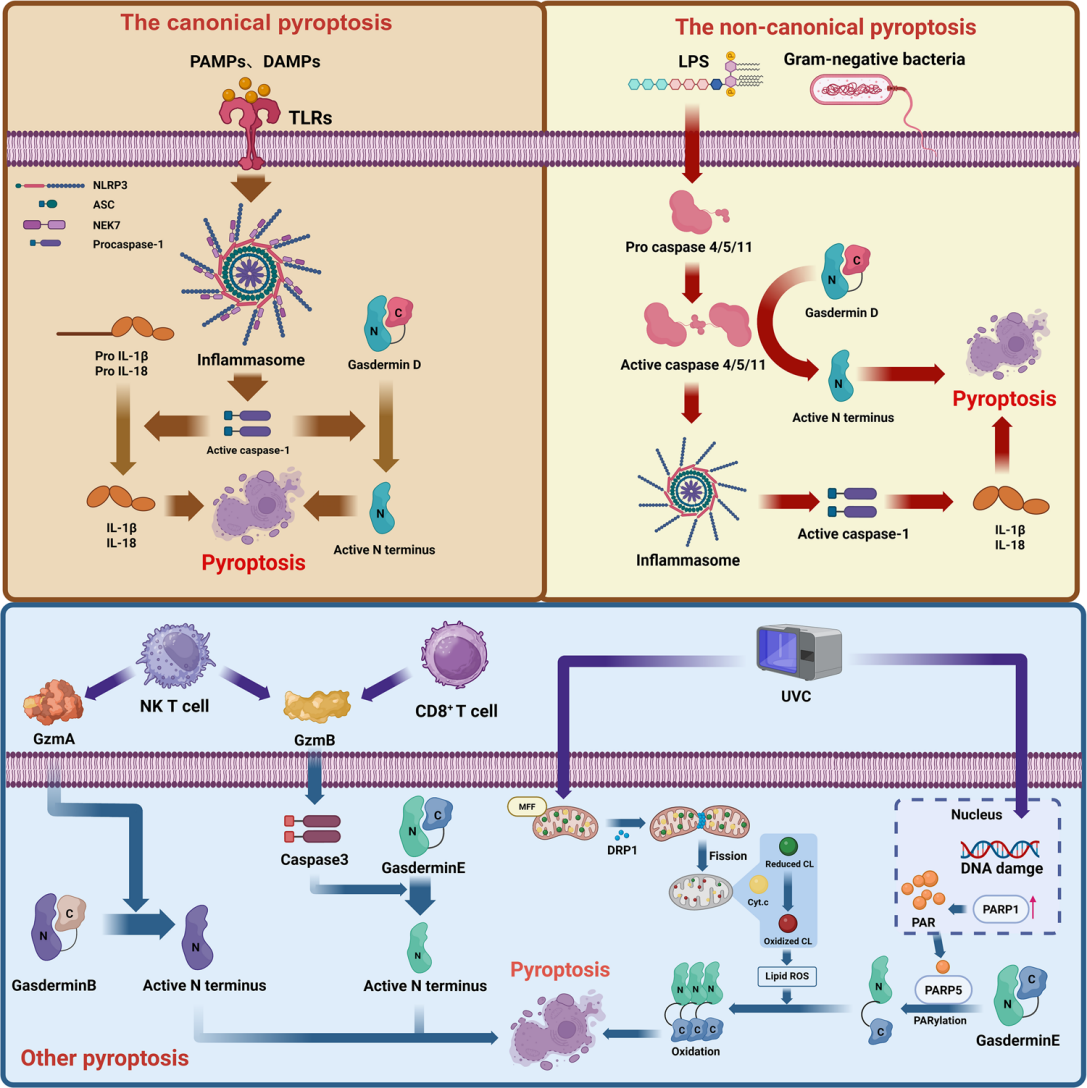
Mechanisms of pyroptosis.(Created by Biorender).

Pyroptosis, a form of programmed cell death, is executed through multiple molecular pathways. These include the classical and nonclassical inflammasome pathways, granzyme-dependent alternative pathways mediated by immune cells and a unique pathway involving full-length GSDME that is triggered by PARylation and oxidative oligomerization independently of proteolytic cleavage. Collectively, these mechanisms illustrate the complex regulatory network governing pyroptosis in immune responses and cellular fate determination (Fig. 2).

#### The canonical inflammasome pathway

In the canonical inflammasome pathway, cells first activate the NLRP3 inflammasome by pathogen-associated molecular patterns or DAMPs^39^. NLRP3 is a cytoplasmic pattern recognition receptor that typically requires two signals for activation: the initial signal (signal 1) and the activation signal (signal 2).

Signal 1 is typically mediated by pattern recognition receptors on the cell surface, such as Toll-like receptors, which upregulate the expression of NLRP3 and pro-IL-1β through the NF-κB signaling pathway. This process ensures that cells have sufficient NLRP3 and precursor cytokines to respond to subsequent stimuli. Signal 2 is triggered by various stimuli, including ATP, uric acid crystals, changes in intracellular potassium ion concentration, mitochondrial damage and the production of ROS. These stimuli cause oligomerization and activation of the NLRP3 protein^40^. Activated NLRP3 interacts with apoptosis-associated speck-like protein (ASC), which binds to NLRP3 through its PYD domain and recruits and activates pro-caspase-1 through its CARD domain^41^. The activation of caspase-1 is a crucial step in the function of inflammasomes. Activated caspase-1 cleaves GSDMD, releasing its N-terminal fragment^42^. The GSDMD-N fragment forms pores on the cell membrane, causing leakage of cellular contents and ultimately leading to pyroptosis. Meanwhile, caspase-1 cleaves pro-IL-1β and pro-IL-18, allowing them to mature into active forms of IL-1β and IL-18. These pro-inflammatory cytokines are released into the extracellular space through membrane pores, further amplifying local and systemic inflammatory responses^6^.

#### The noncanonical inflammasome pathway

The noncanonical inflammasome pathway is an important induction mechanism of pyroptosis. Unlike the canonical pathway, it does not rely on the assembly of conventional inflammasomes, such as NLRP3, but is mediated by the direct activation of caspase-11 (in mice) or caspase-4/5 (in humans)^43,44^. In the noncanonical inflammasome pathway, intracellular lipopolysaccharide (LPS), a cell wall component of Gram-negative bacteria that can directly enter the cytoplasm and bind to caspase-11 or caspase-4/5, is the major activator^45^. This binding does not need to be mediated by conventional pattern recognition receptors but rather by direct interaction of LPS with the CARD structural domain of caspase, leading to oligomerization and self-activation of caspase^46^. Once caspase-11 or caspase-4/5 are activated, they cleave GSDMD, releasing its N-terminal fragment. The GSDMD-N-terminal fragment inserts itself into the cell membrane and forms a pore, increasing the cell membrane’s permeability, leaks cellular contents and ultimately triggers pyroptosis^47^. In addition, activation of the noncanonical inflammasome pathway indirectly activates the NLRP3 inflammasome by inducing potassium efflux and other ion imbalances, thereby promoting caspase-1 activation and pro-inflammatory cytokine release^48^. This cross-activation mechanism further amplifies the inflammatory response. The noncanonical inflammasome pathway plays an important role in the fight against bacterial infections, especially against intracellularly parasitized Gram-negative bacteria^44^. However, its overactivation may also lead to excessive inflammatory responses associated with the pathologic processes of several inflammatory diseases^49^. Therefore, an in-depth study of the regulatory mechanisms of the noncanonical inflammasome pathway is essential for developing new anti-inflammatory therapeutic strategies.

#### Alternative signaling pathways

The alternative signaling pathway is a unique mechanism for triggering pyroptosis and primarily involves the active participation of immune cells, such as cytotoxic T lymphocytes (CTLs), natural killer cells (NK cells) and chimeric antigen receptor T cells (CAR-T cells)^50,51^. These immune cells trigger pyroptosis of target cells by releasing perforin and granzyme. In this pathway, when CTLs and NK cells recognize and bind to a target cell, they release perforin, a protein that forms holes in the target cell membrane. The holes formed by perforin allow granzymes, such as granzyme B (GZMB), to enter the cytoplasm of the target cell. Upon entering the target cell, GZMB cleaves GSDME and releases its N-terminal fragment. The N-terminal fragment of GSDME inserts itself into the cell membrane. It forms a pore, increasing membrane permeability, which triggers the leakage of cellular contents and pyroptosis. In addition, GZMB activates caspase-3, which cleaves GSDME and enhances the pyroptosis effect^52^. This mechanism suggests that the alternative signaling pathway relies on the direct action of granzyme and may also enhance cell death signaling by activating the downstream caspase pathway. The alternative signaling pathway plays a key role in immune monitoring and anti-tumor immunity. By inducing pyroptosis, CTLs and NK cells can eliminate infected and cancerous cells effectively. However, excessive activation of this pathway may trigger autoimmune diseases and tissue damage. Therefore, an in-depth understanding of the molecular mechanisms of alternative signaling pathways is important for developing novel immunotherapeutic strategies.

#### Pyroptosis independent from cleavage mediated by full-length GSDME

Zhou et al. have recently discovered a novel mechanism of pyroptosis^53^. The study indicates that full-length GSDME can trigger cellular death without relying on conventional protease cleavage. High-intensity UVC radiation mediates this process through two key signaling pathways. First, it causes DNA damage, activating PARP1 and PARP5. PARP1 generates cytoplasmic PAR chains that activate PARP5, which PARylates GSDME, releasing it from autoinhibition. Second, UVC triggers lipid peroxidation, generating ROS that oxidatively oligomerize GSDME. This enables its N-terminal fragment to form plasma membrane pores, increasing permeability, and inducing pyroptosis^53^. This mechanism suggests that GSDME can independently mediate pyroptosis through PARylation and oxidative oligomerization without relying on protease cleavage.

### Pyroptosis in IDD

#### ROS and pyroptosis

In IDD, the pathological cascade reaction formed between ROS and pyroptosis is highly complex and plays a key role in driving the onset and progression of IDD^54^. This process results in a massive accumulation of ROS in the hypoxic, hyperacidic and nutrient-poor microenvironment of the IVD, mainly due to mitochondrial dysfunction and abnormal metabolism. Excess ROS induces intracellular changes through various mechanisms; first, it causes mitochondrial damage and contributes to potassium ion efflux, which leads to changes in the intracellular environment^55^. ROS also regulates thioredoxin (TXNIP) release through redox reactions, and these changes ultimately activate the NLRP3 inflammasome^56^. The activation of the NLRP3 inflammasome subsequently recruited and activated caspase-1, which promotes the formation of pores on the cell membrane by cleaving GSDMD. This leads to the leakage of cellular contents and pyroptosis^26^. In addition, ROS and the inflammatory state triggered by pyroptosis promote vascularization and inward nerve growth, creating a vicious cycle of ROS–pyroptosis–inflammation that further exacerbates IDD^57^.

#### Pyroptosis and ECM metabolism

An imbalance between ECM synthesis and degradation is a key pathologic feature in IDD. The normal functioning of IVDs is impossible without the abundant presence of collagen 2 (Col2) and proteoglycans, especially in the NP^58^. MMP (for example, MMP1, MMP3, MMP9, MMP10 and MMP13) and depolymerizing ADAMTS enzymes (for example, ADAMTS-4 and ADAMTS-5) are the major Col2 and proteoglycan-degrading enzymes^59^. Recent studies have found that pyroptosis can stimulate the production of MMP and ADAMTS during the IDD process in vitro and in vivo. In addition, inhibition of NLRP3 inflammasome activity effectively prevented ECM degradation and slowed the progression of IDD. Bromodomain-containing protein 4 (BRD4) plays an important upstream role in regulating the activity of the NLRP3 inflammasome. Studies have shown that JQ1, an inhibitor of BRD4, inhibited the protein-induced activation of NLRP3 inflammasome, suppressed the overexpression of MMP3 and MMP13 and ADAMTS-4 and ADAMTS-5, and increased the levels of Col2 and proteoglycans, effectively slowing down the progression of IDD^60^.

Due to the low oxygen tension level in IVD, NPC mainly relies on anaerobic glycolysis as its main energy metabolism pathway. Under pathological conditions such as ischemia, inflammation and hypoxia, the acid-base balance of the body’s internal environment is disturbed, which leads to the development of tissue acidosis. It has been found that lactate concentration is significantly elevated in myeloid cells, which acts as an extracellular acid to activate acid-sensitive ion channels (ASIC1a and ASIC3), triggering the inward flow of Ca^2+^ (ref. ^61^). Ca^2+^ signaling elevates ROS to activate the NF-κB pathway, promoting NLRP3 inflammasome assembly. NLRP3 activation triggers IL-1β release and pyroptosis, leading to ECM degeneration and accelerating IDD^62^.

#### Pyroptosis and Inflammation

Inflammation plays a crucial role in the IDD process. Studies indicate that pro-inflammatory factors such as TNF and IL-1 are significantly increased in degenerated and herniated human disc tissues. These mediators disrupt disc cell function through various pathways^63^. They can also induce the expression of nerve growth factor, inhibit the transcription of Sox9 and Col2, and modulate the expression level of ADAMTS-4^64^. In addition, pro-inflammatory factors may increase the recruitment and infiltration of immune cells by promoting the secretion of chemokines, exacerbating the degenerative process of the IVD^65^. Propionibacterium acnes activates the NLRP3 inflammasome, upregulating IL-1β and IL-18 expression and inducing NPC pyroptosis. This NLRP3-dependent pathway represents a key mechanism in the inflammatory pathology of IDD^65^.

#### Pyroptosis with vascularization and inward nerve growth

IVDs are the largest avascular tissues in living organisms. During the progression of IDD, IVD tissues gradually become vascularized and innervated, and such changes are positively correlated with the severity of IDD^66^. Vascular endothelial growth factor (VEGF) is a key pro-angiogenic factor that plays an essential role in the formation and development of blood vessels^67^. During IDD, increased expression of IL-1β is closely associated with the upregulation of VEGF and neurotrophic factors (NGF and BDNF), which promote angiogenesis and inward neural growth^68,69^. IL-1β upregulates the expression of multiple inflammatory and catabolic factors and activates the pyroptosis pathway. Consequently, pyroptosis may contribute to IDD by promoting angiogenesis and neural ingrowth, thereby accelerating structural deterioration. Since IL-1β itself is a product of pyroptosis, inhibiting pyroptosis could represent a feasible therapeutic strategy to suppress vascularization and aberrant nerve infiltration in degenerative discs^70^.

## Pyroptosis-based IDD treatment strategies

Pyroptosis-targeted therapies are gaining attention for slowing IDD progression. Various agents, including specific inhibitors and natural compounds, have demonstrated efficacy in vitro, offering promising therapeutic avenues. This section will review such strategies, including natural molecules, drugs, extracellular vesicles (EVs) and novel biomaterials (Table 1).

**Table 1:** Pyroptosis-based therapy in IDD.

Classification	Compound	Experimental models(stimuli)	Function	Signaling pathway	References
Natural molecules	KA	Rat NPC(TBHP); rat NPC(puncture)	PANoptosis ↓, catabolism ↓, ROS↓	TAK1 ↑, PI3K/AKT/NF-κB ↓	^72^
	Morin	Rat NPC(TNF)；mice NPC(spinous process resection)	pyroptosis ↓	TXNIP/NLRP3/caspase-1/IL-1β ↓	^74^
	Paeoniflorin	Rat NPC(puncture)	pyroptosis ↓, catabolism ↓, intracellular calcium concentration ↓	NLRP3/ASC/caspase-1 P20/GSDMD-N ↓	^76^
	Maltol	Rat NPC(IL-1β)；mice NPC(spinal instability)	pyroptosis ↓, catabolism ↓, inflammation ↓	PI3K/AKT/NF-κB ↓, NLRP3 ↓	^78^
Endogenous small molecules	APN	Human NPC(LPS)	pyroptosis ↓, LDH ↓	miR-135a-5p ↑, TXNIP/NLRP3 ↓	^80^
	Kindlin-2	Human NPC(Kindlin-2 deletion)；rat NPC(puncture)	apoptosis ↓, catabolism ↓, inflammation ↓	NLRP3/IL-1β ↓	^82^
	GDF-5	Human NPMSC(LPS)	pyroptosis ↓, chondroid differentiation ↑, inflammation ↓	RhoA ↑	^84^
	MFG-E8	Human NPC(H_2_O_2_)；rat NPC(H_2_O_2_, puncture)	pyroptosis ↓, catabolism ↓, ROS ↓, mitochondrial dysfunction ↓	PI3K/AKT/Nrf2 ↑, TXNIP/NLRP3 ↓	^85^
	BMP7	Rat NPC(STZ)	pyroptosis ↓, inflammation ↓	NLRP3 ↓	^87^
	SIRT1	Human NPC(IL-1β)；rat NPC(puncture)	pyroptosis ↓, mitophagy ↑, ROS ↓	PINK1/Parkin ↑, NLRP3 ↓	^88^
	PDGF-BB	Human NPC(IL-1β)；rat NPC(LSI)	pyroptosis ↓, catabolism ↓, glycolytic enzymes ↑, mitochondrial function ↑	PDGFR-β/TXNIP/NLRP3 ↓	^90^
	Melatonin	Human NPC(IL-1β, TNF, LPS)；rat NPC(puncture)	pyroptosis ↓	IL-1β/NF-κB-NLRP3	^92^
Medicine	Verapamil	Rat NPC(TBHP, puncture)	pyroptosis ↓, apoptosis ↓, ROS ↓	Nrf2 ↑, TXNIP/NLRP3 ↓	^94^
	Rosuvastatin	Rat NPC(TNF, puncture)	pyroptosis ↓, catabolism ↓, senescence ↓	HMGB1-NF-κB ↓	^96^
Stem cell and EVs	hASC-EV	Human NPC(LPS); rat NPC(puncture)	pyroptosis ↓, autophagy ↑, catabolism ↓	TGFβR2 ↓, Akt/mTOR↑	^108^
	hESC-EV	Human NPC(H_2_O_2_)；rat NPC(puncture)	pyroptosis ↓	miR-302c/NLRP3 ↓	^109^
	PRP-EV	Human NPC(TBHP); rat NPC(H_2_O_2_, puncture)	pyroptosis ↓, catabolism ↓, inflammation ↓	MALAT1/microRNA-217/SIRT1 ↑, miR-141-3p/NRF2/KEAP1 ↑	^110,111^
	hucMSC-EV	Human NPC(METTL14 overexpression)	pyroptosis ↓	miR-26a-5p/METTL14/NLRP3/caspase-1 ↓	^112^
	Cavin-2-engineered BMSC-EV	Human NPC(TNF)；rat NPC	pyroptosis ↓, ROS ↓	–	^113^
Bioactive materials	dECM@exo	Rat NPC(puncture)	pyroptosis ↓, catabolism ↓	–	^116^
	dECM/M2-sEVs	Human NPC(H_2_O_2_)；rat NPC(puncture)	pyroptosis ↓, ROS ↓	miR-221-3p/PTEN/NLRP3 ↓	^117^
	siDDIT4@G5-P-HA	Rat NPC(H_2_O_2_, puncture)	pyroptosis ↓, ROS ↓	ROS/TXNIP/NLRP3↓	^24^
	FG@PEV	Rat NPC(LPS + ATP, puncture)	pyroptosis ↓, ROS ↓, catabolism ↓, free fatty acid metabolism ↑	–	^118^
	PEG-PIB	Human NPPC	pyroptosis ↓	COX2/NF-κB/caspase-1 ↓	^119^
	PG@Cu-FP	Rat NPC(H_2_O_2_, puncture)	pyroptosis ↓, ROS ↓, apoptosis ↓, catabolism ↓	Nod-like receptor ↓	^120^
	MCDs	Human NPC(H_2_O_2_)；rat NPC(puncture)	pyroptosis ↓, ROS ↓, catabolism ↓, mitochondrial function ↑	–	^122^

### Natural molecules

#### KA

Kongensin A (KA) is a natural plant product extracted from croton with antinecrotic and anti-inflammatory effects. Studies have shown that it is important in protecting NPCs during IDD^71^. KA upregulates the expression of TGF-β-activated kinase 1 (TAK1) to inhibit PANoptosis (including apoptosis, necroptosis and pyroptosis) in NPCs, thereby reducing oxidative stress damage to cells, restoring mitochondrial function in vitro. In vivo, KA inhibits PANoptosis and promotes ECM regeneration^72^.

#### Morin

Morin, a natural polyphenol from plants, exhibits multiple pharmacological benefits including antioxidant and anti-inflammatory effects^73^. Morin primarily protects NPCs and alleviates IDD by inhibiting the TXNIP-mediated NLRP3–caspase-1–IL-1β signaling pathway. In vitro, it reduces TNF-induced pyroptosis and collagen degradation while upregulating Col2 expression. In addition, morin suppresses pyroptosis by inhibiting NF-κB and caspase family activation. In vivo, it downregulates TXNIP, restores disc height and exhibits notable anti-inflammatory and NPC-protective effects^74^.

#### Paeoniflorin

Paeoniflorin (PF) is a monoterpene glycoside from peony root, exhibiting antioxidant, antidepressant and immunomodulatory effects^75^. In vitro, PF reduces intracellular calcium and lactate accumulation in puncture-injured NPCs, inhibiting the expression of pyroptosis-related proteins. It also mitigates ECM degradation by suppressing MMP3/MMP13 and promoting Col2 and aggrecan. In vivo, PF downregulates pyroptosis-related inflammatory cytokines in degenerated discs, alleviates acidosis, preserves ECM integrity and delays disc height loss, thereby improving cushioning function^76^.

#### Maltol

Maltol, a compound from red ginseng, exhibits anti-inflammatory, antioxidant and anti-aging properties^77^. Molecular docking indicated strong MA-PI3K binding. In vitro, MA binding inhibits PI3K/AKT, suppressing NLRP3 inflammasome activation, pyroptosis-related proteins and IL-1β/IL-18 release. In vivo, MA similarly inhibits PI3K/AKT/NLRP3, reduces pyroptosis, alleviates inflammation, preserves ECM, and delays IDD progression^78^.

### Endogenous small molecules

#### APN

Adiponectin (APN) is a protein secreted by adipocytes, its main functions include anti-inflammatory, anti-apoptotic and enhanced insulin sensitivity^79^. Research has shown that APN expression is reduced in degenerated IVD tissue, and its overexpression can significantly downregulate TXNIP protein associated with pyroptosis, thereby inhibiting the activation of NLRP3 inflammasome and reducing the release of IL-1β and IL-18 in vivo. The miR-135a-5p mediates this process, and upregulating APN helps protect NPCs and slow IDD^80^.

#### Kindlin-2

Kindlin-2 is a focal adhesion protein, along with other focal adhesion proteins such as talin and vinculin. These activate integrins and regulate several basic cellular processes, such as migration and ECM adhesion^81^. Research has shown that kindlin-2 is highly expressed in the NPCs but not in the AF and the CEP. In vitro, kindlin-2 deletion or knockdown induces NPC apoptosis and ECM degradation and activates the NLRP3 inflammasome with increased IL-1β expression, suggesting a cell-autonomous inflammatory-degenerative cycle. In vivo, disc-specific kindlin-2-knockout mice spontaneously develop IDD and exhibit accelerated degeneration under mechanical stress^82^. Although the study did not directly link kindlin-2 to pyroptosis, its role in maintaining disc homeostasis may involve suppressing pyroptosis by inhibiting NLRP3 inflammasome activation.

#### GDF-5

Growth differentiation factor-5 (GDF-5) is a member of the TGF-β/BMP superfamily. GDF-5 functions by binding BMP receptors, activating downstream signaling and regulating cell proliferation and differentiation to promote cartilage and bone formation^83^. Recent studies show that GDF-5 plays a role in IDD by inhibiting pyroptosis. In vitro, GDF-5 mitigates LPS-induced inflammation in NP-derived mesenchymal stem cells (NPMSCs) by reducing TNF and IL-1β, and specifically inhibits pyroptosis via RhoA pathway activation. In vivo, GDF-5 administration attenuates IDD progression, correlating with suppressed pyroptosis and improved disc structure^84^.

#### MFG-E8

Milk fat globule factor-8 (MFG-E8) is an endogenous secreted glycoprotein with beneficial effects in anti-inflammatory, antioxidant and regulation of NLRP3 inflammasome. Exogenous MFG-E8 suppresses H₂O₂-induced oxidative stress, mitochondrial dysfunction, and NLRP3 inflammasome activation by regulating the Nrf2–TXNIP–NLRP3 axis, thereby reducing pyroptosis and ECM degradation in vitro. Administration of MFG-E8 effectively delayed IDD progression in a rat model^85^.

#### BMP7

Bone morphogenetic protein 7 (BMP7) is one of the members of the TGF-β superfamily and is widely used in the treatment of IDD due to its notable impact on cellular synthesis, metabolism and differentiation^86^. BMP7 attenuates IDD by regulating the inflammatory response and pyroptosis. In vitro, BMP7 inhibits NLRP3 inflammasome activation in NPCs, reducing the release of IL-1β, IL-18 and GSDMD-N, mitigating pyroptosis under a diabetic-like microenvironment. In vivo, BMP7 treatment alleviates diabetes-induced IDD, partly through pyroptosis suppression^87^.

#### SIRT1

SIRT1 is a member of the sirtuin family and plays a crucial role as an NAD^+^-dependent deacetylase. Research has shown that SIRT1 plays a key role in inhibiting pyroptosis and improving mitochondrial oxidative metabolism. In vitro, IL-1β induces pyroptosis and mitochondrial dysfunction in NPCs. SIRT1 overexpression activates the PINK1–Parkin pathway, promotes mitophagy, reduces ROS and inhibits NLRP3-mediated pyroptosis. In vivo, SIRT1 activation alleviates IDD in animal models, primarily through pyroptosis suppression via mitochondrial protection^88^.

#### PDGF-BB

Platelet-derived growth factor-BB (PDGF-BB) is a peptide regulator that promotes tissue cell growth^89^. PDGF-BB acts against IDD by promoting ECM synthesis in NPCs and inhibiting pyroptosis. In vitro studies demonstrate that PDGF-BB treatment effectively inhibits IL-1β-induced pyroptosis in NPCs through the PDGFR-β–TXNIP signaling pathway. In vivo studies confirm that PDGF-BB administration alleviates IDD by suppressing pyroptosis and modulating glycolytic metabolism in disc tissues^90^.

#### Melatonin

Melatonin exhibits potent antioxidant and anti-inflammatory properties. It shows notable potential for treating diseases related to oxidative stress and inflammation^91^. Melatonin demonstrates potential for treating IDD due to its anti-inflammatory properties. Studies demonstrate that melatonin directly interrupts the IL-1β–NF-κB–NLRP3 inflammasome positive feedback loop in NPCs, effectively suppressing caspase-1 activation and GSDMD cleavage, thereby inhibiting pyroptosis^92^.

### Medicine

#### Verapamil

Verapamil is a mature treatment for angina pectoris, arrhythmia and cardiomyopathy^93^. However, studies have shown that it also plays a role in IDD by inhibiting oxidative stress and pyroptosis. In vitro evidence indicates that verapamil modulates the Nrf2–TXNIP–NLRP3 signaling axis in NPCs, effectively attenuating NLRP3 inflammasome activation and subsequent pyroptosis through reduction of TXNIP expression and ROS generation. In vivo studies confirm that verapamil ameliorates IDD by suppressing pyroptosis and its associated inflammatory response^94^.

#### Rosuvastatin

Rosuvastatin is a hydroxymethylglutaryl-CoA (HMG-CoA) reductase inhibitor used to treat patients with dyslipidemia. Studies have shown that rosuvastatin prevents coronary artery microembolism-induced cardiac injury by inhibiting NLRP3 inflammasome activation^95^. Chen et al. found that rosuvastatin suppresses TNF-induced NLRP3 inflammasome activation and pyroptosis in NPCs through inhibiting the HMGB1-NF-κB pathway in vitro^96^. In vivo, rosuvastatin treatment effectively attenuates IDD, with histological evidence confirming its role in inhibiting pyroptosis and preserving matrix integrity^96^.

### Stem cells and EVs

Mesenchymal stem cells (MSCs) were first described by Friedenstein in 1968. They belong to a type of adult pluripotent stem cell and are present in almost all tissues and organs^97^. MSCs have notable regenerative ability, anti-inflammatory effect, anti-apoptotic properties, anti-angiogenic ability, anti-fibrotic properties and immune regulatory function^98^. However, MSCs have limitations, such as a low cell survival rate^99^. Due to the unique structure of IVD, the NP tissue is surrounded by a large amount of AF tissue. Therefore, transporting a large number of MSCs to NP tissue inevitably causes damage to the structure of IVD, thereby limiting the therapeutic effect of MSCs. However, EVs derived from MSCs, due to their small size, low immunogenicity and easily obtainable characteristics^100^, have become a promising treatment for IDD. The following introduces EVs.

EVs can be classified based on their cellular origin, biological function or biogenesis. According to their biogenesis, EVs can be classified into three types: exosomes, microvesicles and apoptotic bodies^101^. Most EVs are formed by endosomes generated by endocytosis in cells. These endosomes grow inward to form multivesicular bodies and are ultimately released by fusion with the cell membrane^102^. Exosomes typically have a diameter between 50 and 150 nm and possess various biological properties, including promoting tumor formation and tissue regeneration^103^. Microvesicles are formed by budding outward from the cell membrane, with a diameter between 50 and 1,000 nm, and have been shown to promote and inhibit angiogenesis^104,105^. Apoptotic bodies are formed during the process of cell apoptosis, with a diameter range of 1–5 μm. Their biological characteristics include regulating immune responses and tissue development, similar to other EV subtypes^106^.

Currently, most EVs used for IDD treatment come from MSCs, mainly BM-MSCs and AD-MSCs, but the role of EVs from other tissue sources cannot be ignored^107^. Research has confirmed that EVs can alleviate IDD by inhibiting cell death-related signaling pathways. MiR-155-5p in human adipose tissue stem cell-derived EVs (hASC-EVs) promotes autophagy and reduces pyroptosis by targeting TGFβR2, thereby alleviating IDD^108^. MiR-302c in embryonic stem cell-derived EV (hESC-EV) inhibits NLRP3 inflammasome to alleviate pyroptosis in NPCs^109^. PRP-EV, derived from platelet-rich plasma, regulates SIRT1 expression by delivering MALAT1 as a sponge of miR-217, impairing the NF-κB–NLRP3 pathway and inhibiting the progression of IDD^110^.

Studies have shown that PRP-EV downregulates Keap1 by delivering miR-141-3p, releasing Nrf2 from the Keap1-Nrf2 complex, thereby transferring Nrf2 from the cytoplasm to the nucleus^111^. MiR-26a-5p in hucMSC-EV derived from human umbilical cord MSCs is reduced by METTL14, resulting in decreased m6A methylation of NLRP3 mRNA and reduced binding of IGF2BP2 to NLRP3 protein levels^112^. In the future, EVs from more organizational sources may be seen as potential treatment options for IDD.

In IDD, impaired NPCs exhibit reduced EV uptake efficiency. MSC-derived small EVs mitigate inflammation and suppress pyroptosis via delivery of antioxidants such as Prx2. To counter TNF-impaired uptake, engineered EVs expressing cavin-2 enhance internalization, improving NPC survival and regeneration^113^.

### Bioactive materials

#### Hydrogel

Many traditional therapies are administered orally or intravenously, which may limit their efficacy because drug molecules must cross multiple biological barriers and resist degradation in various chemical environments. Hydrogel, with its ECM-mimetic three-dimensional network and high water content, not only supports essential cellular processes such as migration, adhesion, proliferation and differentiation but also offers unique advantages for IDD therapy. Its high hydration capacity helps restore disc height and mechanical cushioning, while the tunable porous structure facilitates nutrient diffusion and metabolic waste removal in the avascular disc environment. Moreover, hydrogel-based systems can be engineered to provide tailored biomechanical support and deliver bioactive molecules specifically to promote NP regeneration and AF repair, making it a highly suitable biomaterial for addressing the complex challenges of IDD^114^. The mechanical properties of these materials are highly adjustable, making them suitable for the special pressure environment of IVD. Research shows that the hydrogel delivery system implanted in degenerated IVD can effectively prolong the drug release and action time, thus improving the therapeutic effect^115^. These unique characteristics make hydrogel one of IDD repair’s most ideal tissue engineering materials. At present, scientists are exploring injectable hydrogels as a minimally invasive method to delay or even reverse the process of IDD.

Although EVs have shown positive therapeutic prospects, improving the targeting effect and sustained release of EVs is also a topic worth exploring. In the treatment strategy of IDD based on pyroptosis, hydrogel is often used as the carrier of MSC and its EV. Xing et al. designed an adipose-derived MSC exosome coupled with thermosensitive decellularized ECM hydrogel (dECM@exo)^116^. The hydrogel system can form a gel in situ to supplement the loss of ECM in NPC and provide a good environment for the growth of NPC. In addition, the system can continuously release EVs derived from adipose-derived MSCs, balance matrix synthesis and degradation by regulating MMPs, and inhibit pyroptosis by reducing in vitro inflammatory responses^116^. The function of macrophages is primarily influenced by their polarization state, mainly the pro-inflammatory M1 state or the anti-inflammatory M2 state. Zhang et al. prepared a decellularized ECM (dECM) hydrogel (dECM/M2-sEVs) for M2-sEVs sustained release^117^. Delivering miR-221-3p inhibited pyroptosis and inflammatory reaction of NPCs, thereby effectively slowing down IDD and promoting ECM regeneration^117^. Small interfering RNA (siRNA) has been confirmed as an important mediator of RNA interference in mammalian cells, capable of efficiently and specifically targeting mRNA. Therefore, siRNA-based therapy is considered a promising treatment option for IDD. Ma et al. developed siDDIT4@G5-P-HA^24^. It is a three-layer hydrogel, mainly composed of the fifth-generation polyamide (PAMAM), hyaluronic acid and siRNA. PAMAM, as a carrier, has excellent biocompatibility and a positive charge and can effectively adsorb and stabilize siRNA. Hyaluronic acid encapsulates PAMAM, further enhancing biocompatibility and promoting cellular absorption. siRNA specifically targets DNA damage-induced transcript 4 (DDIT4) and blocks the expression of DDIT4, thereby reducing oxidative stress damage to NPC, preventing pyroptosis and inflammatory response and improving the structure and function of IVDs^24^.

FG@PEV It is a new type of hydrogel complex that shows the potential to delay IDD by correcting the metabolic disorder of fatty acids and inhibiting the pyroptosis of NPCs. The preparation process involves the combination of platelet EVs with fibrin to form a hydrogel with good biocompatibility. Research shows that FG@PEV can significantly reduce the expression of NLRP3 and GSDMD, inhibit pyroptosis and maintain the homeostasis of ECM, thereby slowing down IDD. In addition, FG@PEV, by enhancing cellular ROS metabolism, inhibiting inflammatory responses and promoting adipocyte differentiation, can improve the structure and function of IVDs^118^.

#### PEG-PIB

In the combination therapy of hydrogel and stem cells, hydrogel acts as a carrier. Xia et al. proposed a strategy of biomaterial premodification^119^. They developed a new esterase-reactive ibuprofen nano micelle (PEG-PIB) for premodifying embryonic origin NP progenitor cells (NPPCs). PEG-PIB is absorbed by NPPCs through endocytosis, forming premodified NPPCs. This process notable enhances the adaptability of cells in acidic and inflammatory microenvironments and inhibits NPPC pyroptosis through the COX2–NF-κB–caspase-1 pathway. In addition, as a sustained-release system, PEG-PIB continuously releases ibuprofen, reducing the inflammatory response of IVDs. At the same time, PEG-PIB premodified NPCs showed notable functional recovery and tissue regeneration abilities in vitro and in vivo experiments, effectively improving the physical properties of IVDs, such as height and moisture content, and promoting cell proliferation^119^. This biomaterial premodification strategy provides a promising synergistic transplantation method for IDD regeneration.

#### PG@Cu-FP

Zhou et al. designed a multifunctional metal polyphenol nanoparticle: PG@Cu-FP. PG@Cu-FP is composed of copper ions and epigallocatechin acid, with high biocompatibility and low cytotoxicity^53^. During its preparation process, copper ions catalyze the polymerization of epigallocatechin acid, forming nanoparticles and enhancing its specific targeting ability through a pentapeptide (FP) modification composed of phenylalanine(L)–lysine(L)–tyrosine(L)–arginine(L)–cysteine(L) targeting mitochondria. PG@Cu-FP nanoparticles exert potent antipyroptosis effects through multiple synergistic mechanisms. They significantly inhibit NLRP3 and NLRC4 inflammasome activation and reduce caspase-1 activity, thereby preventing GSDMD cleavage and pore formation. Their potent antioxidant capacity enables them to scavenge excess ROS, which simultaneously mitigates mitochondrial dysfunction (a key pyroptosis trigger) and reduces oxidative stress-induced apoptotic signaling. Furthermore, PG@Cu-FP demonstrates anti-apoptotic effects by modulating the Bcl-2/Bax balance and inhibiting caspase-9 activation. This comprehensive cellular protection concurrently reduces pro-inflammatory cytokine release (IL-1β, IL-6) and promotes ECM anabolism. In vivo, administration of PG@Cu-FP in IDD models effectively attenuates IDD. It achieves a satisfactory biological process regulated by multifunctional nanoparticles, inhibits overactive inflammation, delays the process of apoptosis and pyroptosis, and maintains the stability of mitochondrial structure and function^120^.

#### Carbonized Mn-containing nanodots

Catalytic biomaterials include biocompatible materials with catalytic performance, high stability and designability, providing reliable treatment options for various diseases. Nanoenzymes are highly representative catalytic biomaterials that can eliminate excess ROS by simulating the function of endogenous antioxidant enzymes^121^. However, previous studies have focused on the ROS scavenging effect of nanoenzymes and have not studied the specific mechanism. Sun et al. designed carbonized Mn-containing nanodots (MCDs) with remarkable enzyme-mimicking activity, particularly in scavenging ROS^122^. In vitro experiments have shown that MCD has a superior ROS scavenging effect compared with the traditional antioxidant *N*-acetyl-L-cysteine. RNA sequencing showed that MCD downregulated 13 apoptosis-related genes. In the puncture-induced IDD rat model, MCDs reversed the elevation of NLPR3 and ASC, indicating that MCDs delayed IDD by inhibiting NPC pyroptosis^122^.

## Discussion

IDD is a multifactorial disease involving inflammation, oxidative stress, apoptosis, autophagy and more recently, pyroptosis. This review outlines the role and regulation of pyroptosis in IDD and discusses both conventional treatments and emerging biomaterial-based strategies. Pyroptosis, mediated by NLRP3 inflammasome activation and GSDMD-executed pore formation, facilitates the release of pro-inflammatory cytokines, promoting ECM degradation, neurovascular ingrowth and pain. Targeting NLRP3/GSDMD signaling represents a promising therapeutic avenue.

Current biomarkers such as NLRP3, IL-1β and IL-18 lack disease specificity. Future studies should identify IDD-specific markers, such as EV-derived proteins or miRNAs, to improve diagnostics. Although pyroptosis inhibitors show efficacy in animal models, their integration with current conservative or surgical treatments remains underexplored. Combinatorial approaches—using NLRP3 inhibitors post surgery or alongside symptomatic management—may enhance outcomes and delay progression.

The avascular nature of discs impedes drug delivery, urging the development of targeted nanocarriers or biomaterials. Immunogenicity and safety profiling via in vitro and primate models are essential. Low-immunogenicity materials may improve translational potential, advancing pyroptosis-targeted therapies toward clinical application.

## Data Availability

No data was used for the research described in the article.
